# Structural and biochemical characterization of a mitochondrial peroxiredoxin from *Plasmodium falciparum*

**DOI:** 10.1111/j.1365-2958.2006.05303.x

**Published:** 2006-07-21

**Authors:** Ian W Boucher, Paul J McMillan, Mads Gabrielsen, Susan E Akerman, James A Brannigan, Claudia Schnick, Andrzej M Brzozowski, Anthony J Wilkinson, Sylke Müller

**Affiliations:** 1 Structural Biology Laboratory, Department of Chemistry, University of York York YO10 5YW, UK; 2 Institute of Biomedical and Life Sciences, Division of Infection and Immunity and Wellcome Centre for Molecular Parasitology, University of Glasgow Glasgow, UK

## Abstract

*Plasmodium falciparum* possesses a single mitochondrion with a functional electron transport chain. During respiration, reactive oxygen species are generated that need to be removed to protect the organelle from oxidative damage. In the absence of catalase and glutathione peroxidase, the parasites rely primarily on peroxiredoxin-linked systems for protection. We have analysed the biochemical and structural features of the mitochondrial peroxiredoxin and thioredoxin of *P. falciparum*. The mitochondrial localization of both proteins was confirmed by expressing green fluorescent protein fusions in parasite erythrocytic stages. Recombinant protein was kinetically characterized using the cytosolic and the mitochondrial thioredoxin (PfTrx1 and PfTrx2 respectively). The peroxiredoxin clearly preferred PfTrx2 to PfTrx1 as a reducing partner, reflected by the *K*_M_ values of 11.6 μM and 130.4 μM respectively. Substitution of the two dyads asparagine-62/tyrosine-63 and phenylalanine-139/alanine-140 residues by aspartate-phenylalaine and valine-serine, respectively, reduced the *K*_M_ for Trx1 but had no effect on the *K*_M_ of Trx2 suggesting some role for these residues in the discrimination between the two substrates. Solution studies suggest that the protein exists primarily in a homodecameric form. The crystal structure of the mitochondrial peroxiredoxin reveals a fold typical of the 2-Cys class peroxiredoxins and a dimeric form with an intermolecular disulphide bridge between Cys67 and Cys187. These results show that the mitochondrial peroxiredoxin of *P. falciparum* occurs in both dimeric and decameric forms when purified under non-reducing conditions.

## Introduction

Peroxiredoxins (Prx) are ubiquitous peroxidases that reduce reactive oxygen and nitrogen species such as hydrogen peroxide, alkyl hydroperoxides and peroxinitrite using an active-site cysteine residue (Bryk *et al*., 2000; Hofmann *et al*., 2002). The reducing equivalents required for these reactions are provided by thioredoxin, glutaredoxin, alkylhydroperoxide reductase, glutathione or cyclophilin (Lee *et al*., 2001; Rouhier *et al*., 2002; Wood *et al*., 2002; Echalier *et al*., 2005). Prx are generally highly abundant proteins, possibly to compensate for their relatively low catalytic activities compared with those of other peroxidases such as catalase and glutathione peroxidase (Hofmann *et al*., 2002). In addition to their antioxidant functions Prx have roles in signal transduction pathways that use hydrogen peroxide as a second messenger (Wood *et al*., 2003a; Veal *et al*., 2004; Choi *et al*., 2005; Okazaki *et al*., 2005; Rhee *et al*., 2005).

According to their structural and catalytic features, Prx are divided into five classes (Echalier *et al*., 2005). A common characteristic of Prx is the presence of a highly conserved cysteine, the peroxidatic cysteine (Cp), in the N-terminal domain of the protein, which accepts the hydroperoxide substrate and forms a cysteine sulphenic acid (Cys-SOH) intermediate during the catalytic cycle (Wood *et al*., 2003a). In 2-Cys peroxiredoxins, this active-site residue is found in the first turn of an α-helix that is able to unwind to form a disulphide bridge with a second cysteine, the resolving cysteine (Cr), close to the C-terminus of the same or another subunit of the dimeric protein (Evrard *et al*., 2004; Echalier *et al*., 2005; Rhee *et al*., 2005). Oneother striking feature of 2-Cys peroxiredoxins is their alternation between oligomeric states – in the reduced state, typical 2-Cys peroxiredoxins are (α_2_)_5_ decamers and in the oxidized state these decamers are typically destabilized and resolved into homodimers (Wood *et al*., 2002; 2003a, b; Echalier *et al*., 2005). However, among 2-Cys peroxiredoxins from different sources, the tendency to form higher oligomers appears to differ. AhpC from *Salmonella typhimurium*, for instance, has been structurally analysed and both reduced and oxidized states were found to be decameric in the crystal structure. (Wood *et al*., 2002). A recent structure of an oxidized form of AhpC from *Mycobacterium tuberculosis* revealed a dodecamer structure consisting of six, rather than five, dimers arranged as a doughnut-like ring (Guimaraes *et al*., 2005).

The malaria parasite *Plasmodium falciparum* possesses five different peroxiredoxins – two [*P. falciparum* 2-Cys peroxiredoxin (PfTrx-Px1) and *P. falciparum* mitochondrial peroxiredoxin (PfTrx-Px2)] show a high degree of sequence similarity to typical 2-Cys peroxiredoxins (Krnajski *et al*., 2001; Rahlfs and Becker, 2001) and two [*P. falciparum* 1-Cys peroxiredoxin (PfTrx-Px3) and PfAOP (AOP: antioxidant protein)] are 1-Cys peroxiredoxins (Kawazu *et al*., 2000; Krnajski *et al*., 2001; Sarma *et al*., 2005). The fifth is a glutathione peroxidase-like protein [*P. falciparum* glutathione peroxidase-like protein (PfTrx-G1)] that prefers thioredoxin as a reductant (Sztajer *et al*., 2001). PfTrx-G1 has been biochemically characterized and shows catalytic efficiencies comparable to thioredoxin-dependent peroxidases from other sources (*k*_cat_/*K*_m_∼ 10^3^−10^4^ M^−1^ s^−1^) whereas the 2-Cys peroxiredoxin PfTrx-Px1 reduces hydrogen peroxide with a higher catalytic efficiency of 10^6^ M^−1^ s^−1^ (Akerman and Müller, 2003). The biochemical and catalytic features of all other *Plasmodium* peroxiredoxins have not been analysed yet and structural information is available only for PfAOP (Sarma *et al*., 2005). Given that the parasites lack catalase and glutathione peroxidase, it has been suggested that peroxiredoxins are key antioxidants that guarantee parasite survival under enhanced oxidative stress (Müller, 2004). However, there appears to be redundancy among the various Prx in *Plasmodium* as the knockout of PfTrx-Px1 is not lethal although the null mutants show an increased sensitivity towards hydrogen peroxide and peroxinitrite (Komaki-Yasuda *et al*., 2003). On the other hand, interruption of the thioredoxin redox cycle, by disruption of the gene encoding thioredoxin reductase, is fatal for the parasites, suggesting that their thioredoxin-dependent peroxiredoxin-linked antioxidant system plays a crucial function in defence against oxidative insults (Krnajski *et al*., 2002).

Like other eukaryotes, *P. falciparum* needs to avoid oxidative insults to its organelles, the mitochondrion and the apicoplast. The mitochondrial respiratory chain results in the formation of superoxide anions that are dismutated by a mitochondrial superoxide dismutase (SOD; Sienkiewicz *et al*., 2004). The resulting hydrogen peroxide has to be removed to prevent oxidative injury to the organelle and this is likely to be achieved by a mitochondrial peroxiredoxin-linked detoxification system as has been reported for mammals and yeast (Miranda-Vizuete *et al*., 2000; Wheeler and Grant, 2004). A potential mitochondrial peroxiredoxin gene of *P. falciparum* (*pftrx-px2*) has previously been cloned and recombinantly expressed but no further biochemical characterization of the protein was performed (Rahlfs and Becker, 2001). Immunofluorescent studies using polyclonal antibodies raised against the protein suggest a mitochondrial localization (Yano *et al*., 2005). In this study, we report the biochemical and structural characterization of PfTrx-Px2 and identify its mitochondrial reducing partner as well as the localization of both proteins to the mitochondrion in parasites. Our results show that PfTrx-Px2 is a thioredoxin-dependent peroxidase with a preference for H_2_O_2_ and that it structurally belongs to the class of typical 2-Cys peroxiredoxins. These data suggest an important function of the PfTrx-Px2-linked detoxification of reactive oxygen species in the parasite's mitochondrion.

## Results

### Expression of PfTrx-Px2 and *P. falciparum* thioredoxin 2 (PfTrx2)

The deduced amino acid sequence of *pftrx-px2* was analysed by a variety of prediction programmes. PlasMit, MitoProt and TargetP predicted that the protein is mitochondrial and that it possesses an N-terminal mitochondrial targeting sequence. The length of the predicted targeting peptide differed according to the prediction software used. TargetP suggested that the first 19 amino acid residues comprise the mitochondrial targeting peptide whereas MitoProt predicted that the first 27 amino acids of the protein comprise the targeting peptide. Three different constructs were used for expression trials of which only the construct *pftrx-px2-2*, missing the first 19 amino acids, produced appreciable amounts of soluble recombinant protein. Therefore, all further studies were performed with protein recombinantly expressed from construct *pftrx-px2-2*. The protein was purified by chelating chromatography using Ni-NTA agarose (Qiagen).

Analyses of the deduced amino acid sequence of *pftrx2* revealed that the predicted protein possesses an N-terminal extension of 47 amino acids before similarity in alignments with other thioredoxins is exhibited. The prediction programmes used suggested a hydrophobic signal peptide followed by an apicoplast transit peptide, although it was deposited in the NCBI database as a mitochondrial thioredoxin (accession number: AAQ05974). The predicted apicoplast transit peptide encompasses the potential active site motif of this unusual thioredoxin (WCQAC instead of WCGPC) and it is unlikely that the length of the targeting peptide is predicted correctly. Therefore three constructs differing in the length of their N-termini were recombinantly expressed. PfTrx2-S3, lacking the first 47 amino acids was solubly expressed at 30°C for 4 h at a yield of 10 mg l^−1^ bacteria and purified by Ni-NTA agaraose (Qiagen). The other two constructs yielded primarily insoluble recombinant proteins or precipitated during and after purification and thus were excluded from further studies. PfTrx2 was reduced by *P. falciparum* thioredoxin reductase (TrxR) and thus its ability to interact with PfTrx-Px2 could be investigated in the coupled assay system used in this study.

### Catalytic activity of PfTrx-Px2

The activity of PfTrx-Px2 was analysed using different reducing systems. The assays revealed that the enzyme is thioredoxin-dependent and that neither glutathione nor dihydrolipoamide had the capacity to reduce the peroxiredoxin. The catalytic parameters of PfTrx-Px2 differ considerably depending on the thioredoxin used in the assay system. When Trx1 was used as the reductant, hydrogen peroxide and *tert*-butylhydroperoxide were reduced with similar kinetic parameters whereas cumene hydroperoxide was not accepted as a substrate by the peroxidase (Table 1). The data are probably underestimating the true maximal velocity of the enzymatic reaction because the thioredoxin concentrations used were not saturating when analysing the kinetic parameters of the enzyme for the reduction of the hydroperoxides. The reason for this was that increasing Trx1 and Trx2 concentrations led to increased background reaction rates due to the non-enzymatic reduction of the hydroperoxides by the thioredoxins themselves as reported by Akerman and Müller (Akerman and Müller, 2005). Trx2, which potentially is a mitochondrial protein, was found to be a more specific reducing substrate for PfTrx-Px2 with a 10-fold lower *K*_M_ value and a threefold higher catalytic efficiency. To address the differential specificity of PfTrx-Px2 for the two thioredoxins, several surface residues possibly involved in the interaction between the reducing substrate and the peroxidase were altered by site-directed mutagenesis. PfTrx-Px2Mut1 (where Asn62Tyr63, which is adjacent to the active site residue Thr64, is replaced by AspPhe) and PfTrx-Px2Mut2 (where Phe139Ala140 near Arg142 is substituted with ValSer) showed a decrease of *K*_M_ for Trx1 suggesting a role of these residues in distinguishing between the two reducing substrates. The *K*_M_ values of the peroxiredoxin for Trx2 are only marginally changed and this clearly shows that other residues than the two dyads that were analysed in this study are also responsible for the discrimination between the two reducing substrates.

**Table 1:** Kinetic parameters of PfTrx-Px2 and PfTrx-Px2 mutants with Trx1 and Trx2.

	Trx1	Trx2
*K*_*M*_^*app*^ (μM) PfTrx-Px2WT	130.4 ± 37.3	11.6 ± 4.0
*V*_*max*_^*app*^ (μmol min^−1^ mg^−1^)	300.0 ± 54.6	77.2 ± 14.6
*k*_cat_ (s^−1^)	125	32.1
*k*_cat_/*K*_M_ (M^−1^ s^−1^)	0.96 × 10^5^	2.76 × 10^5^
*K*_*M*_^*app*^ (μM) PfTrx-Px2Mut1	86.2 ± 20.1	7.7 ± 2.9
*V*_*max*_^*app*^ (μmol min^−1^ mg^−1^)	316.9 ± 48.2	44.9 ± 7.7
*k*_cat_ (s^−1^)	132	18.7
*k*_cat_/*K*_M_ (M^−1^ s^−1^)	1.5 × 10^5^	2.4 × 10^5^
*K*_*M*_^*app*^ (μM) PfTrx-Px2Mut2	78.8 ± 26.9	12.1 ± 4.1
*V*_*max*_^*app*^ (μmol min^−1^ mg^−1^)	262.0 ± 54.3	73.3 ± 13.2
*k*_cat_ (s^−1^)	109	30.5
*k*_cat_/*K*_M_ (M^−1^ s^−1^)	1.4 × 10^5^	2.5 × 10^5^
*K*_*M*_^*app*^ H_2_O_2_ (μM)	3.6 ± 1.3	2.2 ± 0.4
*V*_*max*_^*app*^ H_2_O_2_ (μmol min^−1^ mg^−1^)	18.0 ± 0.2	35.9 ± 0.2
*k*_cat_ H_2_O_2_ (s^−1^)	7.5	15.0
*k*_cat_/*K*_M_ H_2_O_2_ (M^−1^ s^−1^)	2.0 × 10^6^	6.8 × 10^6^
*K*_*M*_^*app*^*t*-BuOOH (μM)	4.0 ± 1.2	
*V*_*max*_^*app*^*t*-BuOOH (μmol min^−1^ mg^−1^)	19.9 ± 0.2	
*k*_cat_ *t*-BuOOH (s^−1^)	8.3	
*k*_cat_/*K*_M_ *t*-BuOOH (M^−1^ s^−1^)	2.0 × 10^6^	

The parameters displayed in the table are means of 5–10 measurements ± standard deviations. Mut1 and Mut2 are Asp62Ser63 and Val139Ser140 respectively.

### Localization of PfTrx-Px2 and PfTrx2

**Fig. 1:**
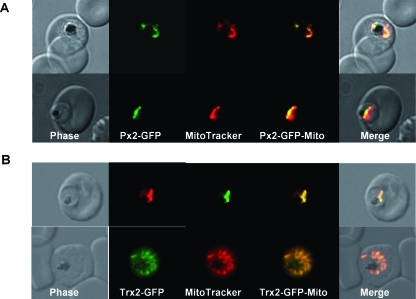
Localization of PfTrx-Px2 and PfTrx2 in *P. falciparum* erythrocytic stages. *P. falciparum* erythrocytic stages were transfected with construct pHH2-Px2-GFP, pHH2-Trx2-GFP leading to the expression of the peroxiredoxin or thioredoxin2 C-terminally fused to green fluorescent protein (GFP). A. The localization of the peroxiredoxin-GFP fusion protein in parasites previously treated with MitoTracker CMX-Ros was analysed by fluorescence light microscopy. Phase, phase contrast of parasitized erythrocytes infected with *P. falciparum* trophozoites; PfTrx-Px2-GFP, parasitized erythrocytes expressing the peroxiredoxin-GFP fusion protein analysed using the FITC channel; MitoTracker, parasitized erythrocytes expressing the peroxiredoxin-GFP fusion protein analysed using the rhodamine channel; PfTrx-Px2-Mito; merge of FITC and rhodamine channels; merge, merge of all images. The images show that the peroxiredoxin-GFP fusion protein is colocalizing with the mitochondrion (stained by MitoTracker). B. The expression of pHH2-PfTrx2 results in the localization of the fusion protein the mitochondrion. Phase, phase contrast of parasitized erythrocytes infected with *P. falciparum* trophozoites; PfTrx2-GFP, parasitized erythrocytes expressing the Trx2-GFP fusion protein analysed using the FITC channel; MitoTracker, parasitized erythrocytes expressing the Trx2-GFP fusion protein analysed using the rhodamine channel; PfTrx2-Mito; merge of FITC and rhodamine channels; merge, merge of all images.

The subcellular localization of PfTrx-Px2 in *P. falciparum* erythrocytic stages was analysed by fusing green fluorescent protein (GFP) to the C-terminus of the full-length protein. The green fluorescence in the parasites expressing the peroxiredoxin-GFP fusion protein was clearly present in an organelle of the parasites that colocalized with the MitoTracker marker (Fig. 1A). This corroborates previous work of Yano *et al*. (2005), who have shown mitochondrial localization of PfTrx-Px2 by immunofluorescence. More importantly, the location of Trx2 was also shown to be mitochondrial, thus suggesting that the protein is the endogenous reducing partner of the mitochondrial peroxiredoxin (Fig. 1B).

### Structure of PfTrx-Px2

**Fig. 2:**
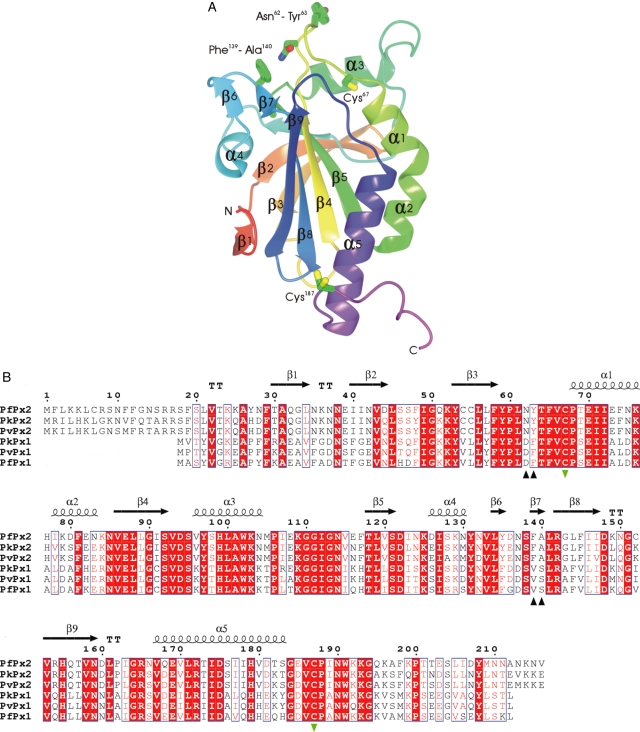
A. Ribbon diagram of the PfTrx-Px2 monomer colour ramped from its N- (orange) to C-terminus (purple) and with elements of secondary structure labelled. The side-chains of the active cysteine residues 67 and 187 (which has a dual conformation) are drawn together with the two pairs of residues (Asn62-Tyr63 and Phe139-Ala140) that were substituted by mutagenesis. B. Amino acid sequence alignment of selected *Plasmodium* 2-Cys peroxiredoxins. Strictly conserved residues are highlighted with red boxes, conservative substitutions are also boxed. Secondary structure elements are shown above the alignment, with TT denoting turns. The active site cysteines are labelled with green inverted triangles. The residues chosen for mutation are indicated by black triangles. This figure was generated using ESPript (Gouet *et al*., 1999). Pf, *P. falciparum*; Pk, *Plasmodium knowlesi*; Pv, *Plasmodium vivax*.

To establish the structure of PfTrx-Px2, crystals of the protein were grown for X-ray diffraction experiments. The structure was solved by the molecular replacement method and refined against X-ray data extending to 1.8 Å resolution as described in *Experimental procedures*. There are two molecules (A and B) in the asymmetric unit of the PfTrx-Px2 crystals. Each PfTrx-Px2 chain contains five α-helices and nine β-strands arranged as a seven-stranded β-sheet and a pair of strands connected by a short turn (Fig. 2). The principal β-sheet has the topology β2-β1-β5-β4-β3-β8-β9, with strands β1 and β8 running antiparallel to the other six strands. Embedded within the structure is a thioredoxin fold encompassing strands β4-β3-β8-β9 of the β-sheet and three of the flanking helices, these being α1, α2, α4 and α5 (Fig. 2). Overall the structure is strikingly similar to those of other 2-Cys peroxiredoxins. PfTrx-Px2 can be superposed onto the dimeric, oxidized rat protein (PDB entry; 1QQ2) and two neighbouring subunits of the decameric, reduced human protein (1QMV) with root mean squared deviation (rmsd) values of 1.3 Å and 1.1 Å, respectively, over 306 and 319 equivalent Cα atoms (Hirotsu *et al*., 1999; Schroder *et al*., 2000).

**Fig. 3:**
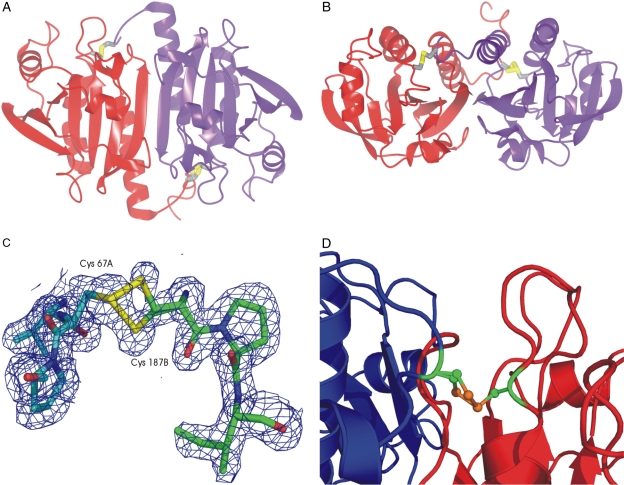
A. The PfTrx-Px2 dimer with subunits coloured in purple and red. This figure was made using the program ccp4mg (Potterton *et al*., 2004). B. Orthogonal view to A to show the intersubunit disulphide bridges. C. Electron density in the vicinity of the intermolecular cystine. The 2*F*_obs_ − *F*_calc_, α_calc_ map is contoured at the 1 σ level. The chains are coloured in cyan and green and the dual conformation of the resolving cysteine from chain B is evident. D. Ribbon diagram showing the active site disulphide in the context of the Cp loop (red) and the C-terminal tail (blue).

The two molecules in the asymmetric unit of the PfTrx-Px2 crystal are related to one another by non-crystallographic twofold symmetry (Fig. 3A and B). The association of this pair of molecules buries approximately 1200 Å^2^ per subunit of what would otherwise be solvent accessible surface area. This accounts for 13% of the total surface area, typical for contact interfaces in dimers (Argos, 1988). In the dimer, the seven-stranded β-sheets from each subunit come together to form an extended 14-stranded β-sheet, with the apposed β9 strands in antiparallel orientation (Fig. 3A).

### Active site and oxidation state

The two active cysteines are each contained in a conserved Val-Cys-Pro segment (Fig. 2), one of which precedes the helix α1 while the other is in the region at the C-terminus of the protein that lacks defined secondary structure. The two active centres in the dimer are separated by approximately 30 Å. Analysis of the PfTrx-Px2 crystal structure clearly reveals that the protein is in the oxidized state. There is an intermolecular disulphide bond between the peroxidatic cysteine, Cys67 of chain A and the resolving cysteine, Cys187 of chain B. This is mirrored by a corresponding disulphide bond between Cys67 of chain B and Cys187 of chain A. It is known that oxidation of other peroxiredoxins is accompanied by conformational changes in the protein in the form of local unwinding of the Cp helix containing the peroxidatic cysteine, Cys67 and a reorganization of the C-terminus (Wood *et al*., 2002). These rearrangements facilitate the close approach of the two cysteines for disulphide bond formation (Fig. 3D). The electron density maps around Cys187 from the B chain indicate the presence of two alternate positions of the sulphur atom, giving rise to a double conformation of the disulphide bond (Fig. 3C) that may be a manifestation of the mobility necessary for the C-terminal cysteine to resolve the peroxidatic cysteine during the reaction mechanism.

There is a conserved glutamic acid residue, Glu70, near to and buried by the disulphide bridge. Its side-chain makes ionic interactions with arginines 142 and 165. The guanidinium group of Arg142 is situated 4.6 Å from the peroxidatic cysteine. This arginine is conserved across 2-Cys peroxiredoxins and it is thought that it may stabilize negative charge that develops on the peroxidatic cysteine in the reaction cycle (Hirotsu *et al*., 1999).

**Fig. 4:**
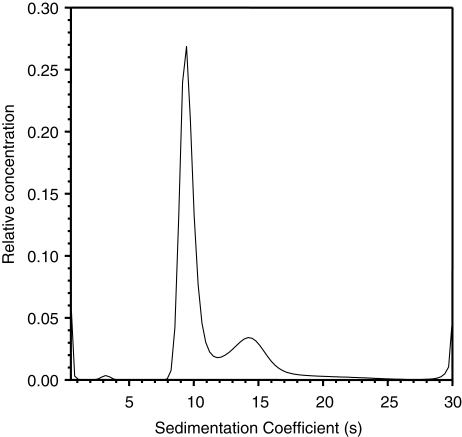
Analytical ultracentrifugation of PfTrx-Px2. A sample of PfTrx-Px2 at an initial concentration of approximately 0.2 mg ml^−1^ was centrifuged at 28 000 r.p.m. A plot of relative concentration versus sedimentation coefficient is shown revealing the presence of peaks at 3.2 S, 9.4 S and 14.2 S. The increases at the limits of the plot indicate there may be some additional aggregates and some very-low-molecular-weight material.

The observation of dimers in the crystal was initially surprising as retention volumes on gel-filtration suggested the predominant species was much larger in size. In the presence and absence of the reducing agent dithiothreitol at 5 mM (DTT), the protein migrated through the column with an apparent molecular weight greater than 200 kDa. The molecular mass of PfTrx-Px2 (in the absence of DTT) was determined more accurately by analytical ultracentrifugation using velocity runs. A plot of relative concentration versus sedimentation coefficient from such a run is shown in Fig. 4 revealing the presence of species with sedimentation coefficients of 3.2 S, 9.4 S and 14.2 S. Assuming equivalent frictional ratios for all species present, these correspond to molecular masses of 50, 230 and 420 kDa respectively. The peak corresponding to 50 kDa appears only at lower concentrations of the PfTrx-Px2 protein. It is likely, allowing for experimental error, that the 420 kDa species is a dimer of the 230 kDa species. As the calculated relative molecular mass of the recombinant PfTrx-Px2 protein is 25.2 kDa, the 230 kDa species corresponds most closely to a nonamer, with octameric to decameric species within the range of experimental error. We interpret the small peak seen at 50 kDa to be the dimeric form of the protein, which was ultimately observed in the crystals.

Our observations are consistent with those of Wood *et al*. (2002), who demonstrated that the oxidized form of 2-Cys peroxiredoxins is an equilibrium mixture of dimers and decamers with the dimeric species observed only at lower concentrations of the protein. These authors crystallized protein purified under non-reducing conditions as an oxidized decamer, in contrast to our observation of oxidized dimers. We observed extensive protein precipitation in the crystallization drops from which our crystals emerged. It is possible therefore that our crystals grew from solutions of much lower protein concentration than the 200 μM originally present in the drop, and at which the dimer form may be more prevalent in solution.

## Discussion

The peroxiredoxin PfTrx-Px2 of *P. falciparum* was previously cloned and expressed in *Escherichia coli* as a full-length protein (Rahlfs and Becker, 2001). Despite the high degree of sequence similarity (51% identity) with PfTrx-Px1, a typical 2-Cys peroxiredoxin of the parasites, the recombinant protein was inactive using *P. falciparum* thioredoxin 1 (PfTrx1) as a reductant in the presence of a variety of hydroperoxide substrates (Rahlfs and Becker, 2001). Analyses of the deduced amino acid sequence of PfTrx-Px2 predicted the presence of a potential mitochondrial targeting sequence at the N-terminus of the protein. Using a variety of prediction programmes, two potential cleavage sites after residues 19 and 27, respectively, were proposed. Expression of both N-terminally truncated PfTrx-Px2 forms in *E. coli* suggests that the first 19 amino acid residues constitute the actual targeting sequence because this construct alone was expressed in a soluble form at an appreciable yield. The localization of the protein was further analysed by expressing the full-length *pftrx-px2* gene fused to GFP in the erythrocytic stages of *P. falciparum* and analysing the localization of fusion protein in the parasites by fluorescent light microscopy. It was clearly targeted to the mitochondrion of the parasites, an observation that was verified by colocalization with MitoTracker. This verifies the localization of PfTrx-Px2 previously shown by immunofluorescent studies (Yano *et al*., 2005). Similarly, the localization of Trx2 was analysed. The Trx2-GFP fusion protein was targeted to the mitochondrion despite the fact that it does not possess a consensus mitochondrial targeting peptide. This has also been observed for *P. falciparum* SOD2 (Sienkiewicz *et al*., 2004) and the precise mechanisms that govern the targeting of these proteins are currently being investigated in more detail.

The structure of PfTrx-Px2 was solved as a dimer in the oxidized form. The active sites of 2-Cys peroxiredoxin contain a disulphide-linked cysteine pair. The two cysteines that form the disulphide arise from a cysteine (Cr) on the C-terminal tail and a cysteine (Cp) preceding helix α1. The PfTrx-Px2 dimer contains a pair of interchain disulphide linkages and thus belongs to the class of typical 2-Cys peroxiredoxins (Wood *et al*., 2003a). The catalytic mechanism of typical peroxiredoxins involves cycling of the active site pair of cysteines between the reduced form and the disulphide-linked oxidized form. The protein is normally maintained in the reduced state by thioredoxin. Under oxidizing conditions, the Cp residue reacts with hydroperoxide substrates to form a cysteine sulphenic acid. In order to prevent over-oxidation of the protein, the Cr residue attacks the oxidized peroxidatic cysteine (Cys-SOH) and a disulphide bridge is formed. During the catalytic cycle it is believed that the protein undergoes structural transitions, typically resulting in the formation of an (α_2_)_5_ decamer in its reduced state, which resolves partially into homodimers at lower concentrations in the oxidized state (Alphey *et al*., 2000; Wood *et al*., 2002). Our data strongly suggest that the mitochondrial peroxiredoxin of *Plasmodium* is predominantly decameric in the oxidized state with a small proportion of dimers that increases as the concentration of the protein lowers. Analysis of the oxidized protein by velocity sedimentation experiments reveals a predominant homodecamer, with a dimeric species at lower concentrations of the protein.

PfTrx-Px2 proved to be a thioredoxin-dependent peroxiredoxin and did not show activity when either glutathione reductase/glutathione or dihydrolipoamide dehydrogenase/lipoamide redox pairs were used in enzymatic assay systems. This is of particular interest because both redox active molecules potentially could act as reducing partners for the peroxiredoxin. Even though the peroxiredoxin is not reduced by these two redox systems, it has been shown previously that thioredoxins can be efficiently reduced by for instance the dihydrolipoamide dehydrogenase/lipoamide redox pair (Akerman and Müller, 2005). Indeed the absence of a mitochondrial thioredoxin reductase from the *P. falciparum* genome implies that lipoic acid (free or protein-bound) might act as a reductant for Trx2 *in vivo* (Müller, 2004). A similar situation has been reported for the peroxiredoxin-linked detoxification in *Mycobacterium*, where it was shown that protein-bound lipoic acid reduces a thioredoxin-like protein leading to the reduction of a peroxiredoxin-like protein and eventually reactive oxygen species (Bryk *et al*., 2002). PfTrx-Px2 is reduced not only by the mitochondrial Trx2 but also by the cytosolic PfTrx1 although their kinetic parameters differed considerably and PfTrx-Px2 clearly preferred thioredoxin-2 to thioredoxin-1 as a electron donor, which is reflected in the distinct apparent *K*_M_ values for both substrates (Table 1). Similarly it was found that in *Drosophila melanogaster* thioredoxin peroxidase-1 is primarily reduced by *Drosophila* thioredoxin-2 rather than *Drosophila* thioredoxin-1 (Bauer *et al*., 2002). This phenomenon was further investigated by mutagenesis studies.

In Fig. 2B the sequences of peroxiredoxins from various *Plasmodium* species are aligned, revealing a high level of sequence conservation with invariant residues at 86 of 189 equivalent positions. The putative mitochondrial enzymes are easily distinguished from their cytosolic counterparts by the presence of the 17 residue extensions at their N-termini. More detailed analysis shows other potential correlations between sequence and subcellular localization. In many positions there are residues that are invariant in the cytoplasmic and mitochondrial subsets but different between them. Some of these differences may be significant for recognition of partner thioredoxin proteins. We looked for such residues situated on the enzyme surface adjacent to the active site that are not involved in dimer formation and may be involved in protein binding. For instance the dyad Asn62Tyr63, which is adjacent to the active site residue Thr64, is replaced by AspPhe in the cytosolic counterpart. Similarly, Phe139Ala140 near Arg142 is substituted with ValSer. Mutant PfTrx-Px2 proteins were made, in which these two surface dyads were exchanged for residues that are more commonly found in cytosolic peroxiredoxins. This resulted in a decrease of the *K*_M_ for Trx1 but had no effect on the kinetic parameters of the enzyme with Trx2. These results suggest that apart from the two dyads that were analysed in this study, other residues are involved in the discrimination between the two reducing substrates. The preference for PfTrx2 suggests that this reductant is the natural substrate of PfTrx-Px2 in contrast to PfTrx1, which, as a cytosolic protein, is unlikely to encounter the mitochondrial peroxidase *in vivo*. The presence of the mitochondrial peroxidase is imperative for the malaria parasite to maintain the integrity of the organelle because it is likely that, through the respiratory chain and the action of mitochondrial SOD, hydrogen peroxide is generated. Therefore we suggest that the mitochondrial peroxiredoxin-linked reduction of H_2_O_2_ is an important line of defence protecting the organelle from oxidative damage. Other redox active molecules like lipoic acid possibly lead to the reduction of Trx2, a hypothesis that will be further tested in future studies. In other organisms, mitochondrial peroxiredoxins and thioredoxins have also been identified and it has been suggested that they play pivotal roles in defence against oxidative stress generated in the organelle (Miranda-Vizuete *et al*., 2000; Wheeler and Grant, 2004). Recently it was reported that the expression of a mitochondrial peroxiredoxin in *Leishmania donovani* protects the parasites against hydrogen peroxide-induced cell death (Harder *et al*., 2006). Further experiments dissecting the precise roles of the peroxiredoxin-linked system using reverse genetics approaches will help to gain a better understanding of the mechanisms that the parasites have employed to protect themselves against fatal oxidative damage.

## Experimental procedures

### Materials

*Plasmodium falciparum* TrxR and PfTrx1 were generated as described in the studies by Gilberger *et al*. (1997) and Akerman and Müller (2003). *P. falciparum* glutathione reductase (GR) was expressed and purified as described previously (Gilberger *et al*., 2000). Hydrogen peroxide, *tert*-butylhydroperoxide and cumene hydroperoxide were from Sigma-Aldrich. The *P. falciparum* expression plasmid pHH2 (Crabb *et al*., 2004) was a kind gift from Professor Alan Cowman, Melbourne, Australia.

### Sequence analyses of PfTrx-Px2 and PfTrx2

The deduced amino acid sequences of PfTrx-Px2 (accession number: AAK20024) and PfTrx2 (accession number: AAQ05974) were analysed for the presence of N-terminal targeting sequences using SignalP (Nielsen *et al*., 1997), TargetP (Emanuelsson *et al*., 2000), MitoProt (Claros and Vincens, 1996), PlasMit (Bender *et al*., 2003) and PlasmoAP (Foth *et al*., 2003). Nucleotide and protein sequence analyses were performed using Vector NTI (Invitrogen). Alignments of the *P. falciparum*-translated *pftrx-px2* and *pftrx2* genes with those of other species were created using ClustalW and were manually adjusted.

### Cloning and expression of PfTrx-Px2 and PfTrx2

*Plasmodium falciparum pftrx-px2* full-length cDNA was amplified from genomic DNA as described previously (Rahlfs and Becker, 2001) using the sense oligonucleotide PfTrx-Px2-S1 5′-GCGCCATATGATGTTTTTAAAAAAACTGTG C-3′ and the antisense oligonucleotide PfTrx-Px2-AS1 5′-GCGCCTCGAGTTACAACTTTGATAAATATTCAC-3′ containing NdeI and XhoI restriction sites respectively. The polymerase chain reaction (PCR) fragment amplified using *Pfu* polymerase (Invitrogen) was cloned into TOPO-blunt (Invitrogen) and subcloned into pJC40 (Clos and Brandau, 1994) after its nucleotide sequence was confirmed. The latter directs expression of the protein with an N-terminal (His)_10_ tag to facilitate purification of the recombinant protein. From the full-length *pftrx-px2* PCR product, two additional constructs were amplified, which lacked coding sequence for the first 19 or 27 amino acid residues respectively. The oligonucleotides used to amplify PfTrx-Px2-S2 were sense 5′-GCGCCATATGTCGCTAGTGACAAAGAAGGC-3′ and antisense oligonucleotide PfTrx-Px2-AS1.

The full-length cDNA of *pftrx2* was amplified using the oligonucleotides PfTrx2-S1 5′-GCGCCATATGAAGAAGTATATATTTTTCTTTCTC-3′ and PfTrx2-AS1 5′-CGCGCTCGAGTTATAAATGTTTTTTAATTAATGC-3′ containing NdeI and XhoI restriction sites. The PCR product was cloned into TOPO-blunt and used as template for amplification using PfTrxs-S3 5′-GCGCCATATGTTTAAAAAAGTACCAAGATTACAACAAAATGG-3′ together with PfTrx2-AS1 to produce constructs that confer the recombinant expression of PfTrx2 lacking the first 47 amino acid residues. All PCR fragments were cloned into TOPO-blunt and once their nucleotide sequences were confirmed, all three constructs were subcloned into pJC40 as described above for the PfTrx-Px2 expression fragments.

The nucleotide sequences were determined by automated sequencing (University of Dundee Sequencing Service). Optimal expression conditions were determined and PfTrx-Px2 was subsequently expressed over 18 h at 20°C in *E. coli* BLR (DE3) and PfTrx2 was expressed over 4 h at 30°C. Soluble expressed recombinant proteins were purified via Ni-NTA agarose (Qiagen) and analysed by SDS-PAGE.

### Mutational analyses of PfTrx-Px2

Site-directed mutagenesis of two dyads close to the active site of PfTrx-Px2 was performed using the quick-change mutagenesis kit (Stratagene). Residues asparagine 62 and tyrosine 63 were substituted with aspartate and phenylalanine using the mutagenic oligonucleotides 5′-GAAATACTGTTGTTTGTTATTTTATCCATTA**GATTTT**ACCTTCGTATGTCCAACAG-3′ and 5′-CTGTTGGACATACGAAGGTA**AAATCT**AATGGATAAAATAACAAACAACAGTATTTC-3′. The resulting mutant protein was named PfTrx-Px2Mut1 and was expressed as described above. Residues phenylalanine 139 and alanine 140 were exchanged by valine and serine using the oligonucleotides 5′-CTAAAAATTATAATGTACTTTATGATAATTCT**GTTTCT**TTAAGAGGTTTATTTATTATTGATAAAAATGG-3′ and 5′-CCATTTTTATCAATAATAAATAAACCTCTTAA**AGAAAC**AGAATTATCATAAAGTACATTATAATTTTTAG-3′. Letters in bold face define the mutated residues. The mutant protein was called PfTrx-Px2Mut2 and recombinant expression and purification was performed as described above. Both mutations were verified by nucleotide sequencing (MWG Biotech).

### Enzymatic assays

The catalytic activities of PfTrx-Px2 were analysed using a stopped-flow rapid kinetics device (SFA-20, Hi-Tech Scientific) attached to a spectrophotometer (Shimadzu UVPC 2501) to determine the initial rates of the peroxidase reaction as previously described for *Toxoplasma gondii* Trx-Px2 (Akerman and Müller, 2005). The reaction mix contained 100 mM HEPES pH 7.6, 1 mM EDTA, 10 μg *P. falciparum* thioredoxin reductase, 0.1 μM PfTrx-Px2, 200 μM NADPH, varying concentrations of *P. falciparum* cytosolic thioredoxin (5–100 μM) or PfTrx2-S3 (1–20 μM) at a constant concentration of 20 μM hydrogen peroxide or varying concentrations of hydrogen peroxide (3–40 μM), *tert*-butyl hydroperoxide (3–40 μM) and cumene hydroperoxide (1–100 μM) at a constant concentration of 10 μM or 8 μM of PfTrx1 and PfTrx2 respectively. The decrease in absorbance at 340 nm due to NADPH oxidation was determined over 30 s with time points taken every 10 ms. Assays were performed at 25°C. The linear rates were determined and fitted to the Michaelis–Menten equation using Graphit 5.0 (Erithracus).

In additional assays we examined whether glutathione or dihydrolipoamide can serve as reductants for PfTrx-Px2. The glutathione reduction assay consisted of 100 mM HEPES pH 7.6, 1 mM EDTA, 200 μM NADPH, 1 mM glutathione, 5 μg of *P. falciparum* GR, 0.4 μM PfTrx-Px2 and 20 μM H_2_O_2_. The dihydrolipoamide reduction assay consisted of 100 mM HEPES pH 7.6, 1 mM EDTA, 1 mM dihydrolipoamide, 200 μM NADH, 5 μg of dihydrolipoamide dehydrogenase (Sigma), 0.4 μM PfTrx-Px2 and 20 μM H_2_O_2_. The change in absorbance in both assay systems was followed spectrophotometrically at 340 nm.

### Gel filtration and analytical ultracentrifugation

PfTrx-Px2 was analysed by gel filtration on a Sephadex S-200 column (1.6 × 60 cm) previously equilibrated at a flow rate of 1 ml min^−1^ with 50 mM sodium phosphate buffer, pH 7.6 containing 150 mM NaCl. Two milligrams of the Ni-NTA purified protein was applied to the column at a flow rate of 1 ml min^−1^ in the same buffer using an FPLC system (Amersham Biosciences).

Sedimentation velocity experiments were performed in a Beckman XL/I analytical ultracentrifuge using Beckman cells with 12 mm path-length double sector charcoal-filled Epon centrepieces and sapphire windows in an AN-60Ti rotor. Protein samples (at approximate concentrations of 0.9 mg ml^−1^ and 0.2 mg ml^−1^ in 0.42 ml) were prepared in 50 mM Tris pH 7.5, 100 mM NaCl and centrifuged at 20 000 and 28 000 r.p.m. over 7.5 h at 20°C along with buffer samples as references. Sedimentation was observed by scanning the absorbance at 280 nm at 3 min intervals until the plateau region disappeared. Data were analysed with the program sedfit (Schuck, 2000) using a simple c(s) model (distribution of sedimentation coefficients without assumption of size or number of species) then transformed to c(M) (distribution of molecular weight). Molecular weights were calculated using the program sednterp (Laue *et al*., 1992).

### Generation of PfTrx-Px2- and PfTrx2-GFP fusion proteins

The full-length coding region of *pftrx-px2* was amplified by reverse transcription (RT)-PCR using the sequence-specific oligonucleotides 5′-GCGCAGATCTATGTTTTTAAAAAAACTGTGC-3′ and 5′-GCGCCCTAGGCAACTTTGATAAATATTCAC-3′ containing BglII and AvrII restriction sites and genomic DNA of *P. falciparum* as a template. The PCR fragments were cloned into TOPO-blunt (Invitrogen) and their nucleotide sequences determined as described above. Subsequently the PCR product was subcloned in frame with GFP into pHH2 previously digested with BglII and AvrII. Similarly the full-length *trx2* open reading frame was amplified by RT-PCR using the sequence-specific oligonucleotides 5′-GCGCAGATCTATGAAGAAGTATATA TTTTTCTTTCTC-3′ and 5′-CGCGCCTAGGTAAATGTTTTTTAATTAATGC-3′ and the resulting PCR fragment was cloned into the BglII and AvrII digested pHH2 plasmid as described above. The plasmid also carries the human *dhfr* gene for selection with WR99210. Transfection of both constructs into *P. falciparum* D10 parasites was carried out by electroporation as previously described (Wu *et al*., 1996). Transfected cell lines were maintained under selection by supplementing media with 5–100 nM WR99210.

### Fluorescence microscopy

Parasites expressing PfTrx-Px2-GFP or PfTrx2-GFP were treated with MitoTracker Red CMX Ros according to Wrenger and Müller (2004) before they were analysed by fluorescence microscopy (Zeiss Axioplan 2 equipped with an HBO100 digital camera).

### Crystallization

Automated crystallization screening was carried out by sitting-drop vapour diffusion in 96 well plates (Greiner) using a Mosquito nanolitre pipetting robot (TTP labtech) and screens from Hampton Research (Crystal Screen, Crystal Screen 2 and Index). Each crystallization drop was made by mixing 150 nl of protein solution at 10 mg ml^−1^ and 150 nl of reservoir solution. PfTrx-Px2 crystallized in 3–4 days from drops containing a large amount of insoluble precipitate from a reservoir solution containing 0.2 M ammonium sulphate, 0.1 M Bis-Tris, pH 6.5 and 25% (w/v) PEG 3350.

### Data collection and processing

Native diffraction data were collected on beamline ID23-1 using radiation with a wavelength of 0.9795 Å at the European Synchrotron Radiation Facility (ESRF). A single crystal was mounted in a rayon loop and transferred to a solution of the crystallization mother liquor containing 15% glycerol and then rapidly cooled in liquid nitrogen. Diffraction data were recorded on an ADSC Quantum 4 CCD detector. Data were processed using MOSFLM and SCALA within the CCP4 program suite (Collaborative Computational Project, 1994). Data collection and refinement statistics are presented in Table 2.

**Table 2:** X-ray data collection and refinement statistics.

	PfTrx-Px2 (PDB code 2C0D)
Data collection		
X-ray source	ESRF Beamline ID23-1
Wavelength (Å)	0.9795
Collection temperature (K)	100
Resolution range (Å)	55.22 – 1.78
Space group	*C*2
Unit-cell parameters (Å,°)	*a* = 104.623, *b* = 76.534, *c* = 60.154 β = 113.34
Matthews coefficient/solvent content (Å^3^. Da)	2.4/42.7
Number of unique reflections	41 762
Completeness (%), overall/outer shell^a^	100 (100)
Redundancy, overall/outer shell^a^	3.61 (3.58)
*I*/σ(*I*), overall/outer shell^a^	13.45 (2.58)
*R*_merge_^b^ (%), overall/outer shell^a^	9.0 (25.0)
Refinement and model statistics	
*R*-factor^b^ (*R*-free^d^)	0.167 (0.203)
Reflections (working/free)	39 652/2106
Outer shell *R*-factor^b^ (*R*-free^d^)	0.198 (0.233)
Outer shell reflections (working/free)^e^	2934/151
Molecules/asymmetric unit	2
Number of protein non-hydrogen atoms	2780
Number of water molecules	280
R.m.s. deviation from target^f^	
Bond lengths (Å)	0.016
Bond angles (°)	1.505
Average *B*-factor (Å^2^)	15.29
Ramachandran plot^g^	89.3/10.0/0.6

a. Data in parentheses correspond to data for outer shell 1.88 – 1.78 Å.

b. R-merge = Σ_hkl_Σ_I_|I_i_ − <I>|Σ_hkl_Σ_I_<I> where I_i_ is the intensity of the ith measurement of a reflection with indexes hkl and <I> is the statistically weighted average reflection intensity.

c. R-factor = Σ||F_o_| − |F_c_||/Σ|F_o_| where F_o_ and F_c_ are the observed and calculated structure factor amplitudes respectively.

d. R-free is the R-factor calculated with 5% of the reflections chosen at random and omitted from refinement.

e. Outer shell for refinement corresponds to 1.826 – 1.78 Å.

f. Root-mean-square deviation of bond lengths and bond angles from ideal geometry.

g. Percentage of residues in most-favoured/additionally allowed/generously allowed regions of the Ramachandran plot, according to PROCHECK.

### Structure solution and refinement

The structure of PfTrx-Px2 was determined by molecular replacement using the program molrep (Vagin and Teplyakov, 1997) and the co-ordinate set for the tryparedoxin from *Crithidia fasciculata* 1E2Y as the search molecule. All data were used in both rotation and translation calculations. A model was built in the program Arp/wARP (Perrakis *et al*., 1999) and refinement calculations were performed using REFMAC5 (Murshudov *et al*., 1997) interspersed with sessions of manual modelling using COOT (Emsley and Cowtan, 2004). The electron density maps were generally of very good quality enabling confident modelling of residues 21–188 of chain A and 21–195 of chain B. The N-terminal histidine tag and residue 20 of both chains, residues 189–216 of chain A and residues 196–216 of chain B were not defined by the maps, and they were assumed to be disordered. Equivalent Cα atoms from chains A and B can be superposed by least squares methods to give a positional rmsd of 0.3 Å. Refinement and model statistics are presented in Table 2.
